# A transcriptomic profile of topping responsive non-coding RNAs in tobacco roots (*Nicotiana tabacum*)

**DOI:** 10.1186/s12864-019-6236-6

**Published:** 2019-11-14

**Authors:** Xi Chen, Shuo Sun, Fangjie Liu, Enhui Shen, Lu Liu, Chuyu Ye, Bingguang Xiao, Michael P. Timko, Qian-Hao Zhu, Longjiang Fan, Peijian Cao

**Affiliations:** 1 Institute of Crop Science, Zhejiang University, Hangzhou, 310058 China; 2 Research Center for Air Pollution and Health, Zhejiang University, Hangzhou, 310058 China; 3 Key Laboratory of Tobacco Biotechnological Breeding, Yunnan Academy of Tobacco Agricultural Sciences, Kunming, 650021 China; 4 Department of Biology, University of Virginia, Charlottesville, VA 22904 USA; 5 CSIRO Agriculture and Food, GPO Box 1700, Canberra, ACT 2601 Australia; 6 Zhengzhou Tobacco Research Institute of CNTC, Zhengzhou, 450001 China

**Keywords:** Co-expression network, Circular RNA, Long non-coding RNA, microRNA, *Nicotiana tabacum*, Topping, Transcriptomic profile

## Abstract

**Background:**

**N**on-coding RNAs (ncRNAs), including microRNAs (miRNAs), long ncRNAs (lncRNAs) and circular RNAs (circRNAs), accomplish remarkable variety of biological functions. However, the composition of ncRNAs and their interactions with coding RNAs in modulating and controlling of cellular process in plants is largely unknown. Using a diverse group of high-throughput sequencing strategies, the mRNA, miRNA, lncRNA and circRNA compositions of tobacco (*Nicotiana tabacum*) roots determined and their alteration and potential biological functions in response to topping treatment analyzed.

**Results:**

A total of 688 miRNAs, 7423 non-redundant lncRNAs and 12,414 circRNAs were identified, among which, some selected differentially expressed RNAs were verified by quantitative real-time PCR. Using the differentially expressed RNAs, a co-expression network was established that included all four types of RNAs. The number of circRNAs identified were higher than that of miRNAs and lncRNAs, but only two circRNAs were present in the co-expression network. LncRNAs appear to be the most active ncRNAs based on their numbers presented in the co-expression network, but none of them seems to be an eTM (endogenous Target Mimicry) of miRNAs. Integrated with analyses of sequence interaction, several mRNA-circRNA-miRNA interaction networks with a potential role in the regulation of nicotine biosynthesis were uncovered, including a QS-circQS-miR6024 interaction network. In this network miR6024 was significantly down-regulated, while the expression levels of its two targets, circQS and its host gene *QS*, were sharply increased following the topping treatment.

**Conclusions:**

These results illustrated the transcriptomic profiles of tobacco roots, the organ responsible for nicotine biosynthesis. mRNAs always play the most important roles, while ncRNAs are also expressed extensively for topping treatment response, especially circRNAs are the most activated in the ncRNA pool. These studies also provided insights on the coordinated regulation module of coding and non-coding RNAs in a single plant biological sample. The findings reported here indicate that ncRNAs appear to form interaction complex for the regulation of stress response forming regulation networks with transcripts involved in nicotine biosynthesis in tobacco.

## Background

Non-coding RNAs (ncRNAs) consist of multiple classes of RNA molecules that are generated by genomic transcription but are not translated into proteins. They include microRNA (miRNA) with ~ 20–24 nucleotides, long ncRNA with a length > 200 bases, and circRNA that is a covalently closed RNA molecule without 5′ cap and poly-A tail formed by back splicing of precursor RNA derived from exons (exonic circRNA), introns (intronic circRNA), or intergenic regions [1–4]. Many studies have looked into the crosstalk between different types of RNA molecules [1, 5, 6]. For example, lncRNA and circRNA may function as miRNA sponges to modulate the activities of both miRNA and its target genes [4, 7]. Noncoding endogenous target mimicries (eTMs) for diverse miRNAs have been identified in *Arabidopsis thaliana*, *Oryza sativa* and *Glycine max*, and some of them have been shown to regulate plant development by repressing miRNA function [8–10]. Wang et al. (2017) identified six differentially expressed wheat circRNAs that respond to dehydration stress and play a role of putative miRNA sponges [11]. Increased evidences for broad functionality of ncRNAs and demonstration of their changing composition and interactions with mRNAs under different conditions in cells well illustrate the crosstalk between different types of RNAs and its importance in plant cellular metabolism. However, no attempt has been made to profile the four types of RNAs and to uncover their interactions in a single plant organ, such as tobacco roots producing nicotine.

The pyridine alkaloid nicotine is the signature compound of *Nicotiana* species, especially in tobacco, responsible for the addiction of smoking and functioning as one of the most effective plant defense metabolites in nature. In tobacco, nicotine is exclusively synthesized in young root tips and subsequently transported to leaves. Nicotine biosynthesis can be induced through topping for tobacco plants self-protection and significant increase of nicotine content can be observed in the topped tobacco plants [12]. The genes involved in nicotine biosynthesis and regulation, such as those encoding quinolinate synthase (QS), quinolinic acid phosphoribosyl transferase (QPT) and putrescine methyltransferase (PMT) have been intensively studied [12–15]. Transcription factors involved in the regulation of nicotine biosynthesis have also been reported, for example, *NIC1/NIC2* was shown to be a cluster of *AP2/ERFs* mediates nicotine formation [16]. A few studies have also investigated the composition and function of ncRNAs in tobacco, with the first topping-responsive miRNA identified in 2011 [17]. Shortly after, Tang et al. (2012) predicted 59 novel tobacco-specific miRNAs using small RNAs from roots of wounding- and topping-treated tobacco plants [18]. With the advent of genomic sequencing techniques and high efficiency bioinformatic analysis, genome-wide scan for ncRNAs has become feasible. Gao et al. (2015) performed genome-wide identification of miRNAs after the releasing of the tobacco genome in 2014 and identified 165 miRNAs, including miRNA members from 55 conserved and 50 novel families, in leaves, stems and roots [19]. Recently, Li et al. (2018) identified 5206 tobacco lncRNAs based on RNA sequencing data, with 565 involved in nematode stress response [20]. ncRNAs have also been implicated in regulation of nicotine biosynthesis in tobacco. For instance, miR164 has been demonstrated to be a negative regulator of *NtNAC-R1*, a unique tobacco NAC (for NAM, no apical meristem) transcription factor responsive to topping and resulted in increase of lateral roots and nicotine contents [21]. In our previous study, a new miRNA, nta-miRX27, was shown to target *QPT2*, one of the key genes involved in nicotine biosynthesis; furthermore, targeting of *QPT2* by nta-miRX27 is attenuated by nta-eTMX27, a lncRNA functioning as an endogenous target mimicry (eTM) to compete against nta-miRX27 for *QPT2* [22]. These results led us to conclude that nicotine biosynthesis is regulated by both miRNAs and lncRNAs. Nevertheless, it is still unclear whether or not circRNAs are also one of the components of the RNA network related to nicotine biosynthesis in tobacco roots.

In this study, four types of RNA molecules (mRNA, miRNA, lncRNA, circRNA) in a single plant organ using high-throughput sequencing strategies were simultaneously characterized for the first time. On the base of identification of the four types of RNAs, their expression levels and abundance were quantified and compared based on a unified normalization approach. A co-expression network for the four types of RNAs was established and a few mRNA-circRNA-miRNA interaction networks were identified, including QS-circQS-miR6024, involved in the regulation of nicotine biosynthesis. The results presented in this study provide launch-pad for further functional characterization of both coding and non-coding RNAs in nicotine metabolism in tobacco.

## Results

### Sequencing and identification of four types of RNAs from a single organ: tobacco roots

*Nicotiana tabacum* cv. K326 was selected for transcriptomic (both coding and noncoding RNAs) profiling. RNA-seq, circRNA-Seq, small RNA-Seq and ssRNA-Seq were performed using roots from both topping-treated (i.e., removal of the floral head and upper young leaves) and untreated tobacco plants. In total, 102.66 Gb of mRNA, 148.86 Gb of circRNA, 25.1 Gb of small RNA and 71.46 Gb of lncRNA raw data were generated (Table 1). The number of the four types of RNA molecules identified and used in this study were shown in Table 2. More than 90% of the 85,570 gene models annotated by Sierro (2014) were supported by transcripts found in our RNA-seq data, with 77,849 and 78,467 genes expressed in the topping-treated and untreated roots, respectively. In this study, a total of 688 miRNAs were inferred, with 268 conserved miRNAs from 38 families and 420 novel miRNAs from 393 families (Additional file 1: Figure S1A). Collected with miRNAs deposited in the miRBase (http://www.mirbase.org, V22) and the ones identified by Li (2015), the total number of tobacco miRNAs analyzed in this study increased to 981 (Table 2). The majority predicted targets of the conserved miRNAs were transcription factors (TF). For example, miR156 and miR167 were predicted to target to squamosa promoter-binding-like (*SPL*) and auxin response factor (*ARF*), respectively, same as that reported in *Arabidopsis thaliana* [23, 24]. miR166 targeting HD-Zip transcription factors found in *Brassica napus* was also identified in tobacco roots [25]. For lncRNAs, in order to have an as complete as possible repository to be used in further analysis, the 6 poly(A) RNA-seq and the 6 ssRNA-seq datasets generated in this study and 82 poly(A) RNA-seq datasets downloaded from NCBI were used for lncRNA identification. As a result, 3635 (3688 transcripts) and 4121 non-redundant lncRNA loci (5352 transcripts) were identified from ssRNA-seq and poly(A) RNA-seq datasets, respectively (Additional file 2: Figure S2A). Only about 10% of the lncRNA loci were identified in both datasets (Additional file 2: Fig. S2B). A much higher proportion of single exon transcripts was found in the ssRNA-seq datasets (86.9%, 3206 transcripts) compared to the poly(A) RNA-seq datasets (4.0%, 214 transcripts) (Additional file 2: Figure S2C). These results confirmed our previous observation in oilseeds [26], suggesting that it is necessary to use both library creating and sequencing methods in lncRNA identification in order to increase the number of lncRNAs identified. circRNA is an emerging star of ncRNA in plants. By integrating the prediction results from three bioinfomatic tools (CIRI, find_circ and PcircRNA-finder), 13,957 circRNA transcripts generated from 8244 host genes were identified. Most of which (11,782, 84.42%) were generated from exons of protein-coding genes, only 1085 (7.77%) were intergenic circRNAs and 7.81% were intronic circRNAs (Additional file 3: Figure S3A). Only around 5% circRNAs were detected by all three tools (Additional file 3: Fig. S3B) probably due to variable sensitivity of the prediction tools towards different types of circRNAs [27]. The general features of the identified tobacco circRNAs consist with those identified in other plants [28, 29], such as more than 80% of exonic circRNAs harbored 1–4 exons (Additional file 3: Figure S3C) and most genes produced only one isoform of circRNAs (Additional file 3: Fig. S3D). However, compared to other plants, tobacco has more circRNAs containing more than 7 exons.

**Table 1:** Summary of raw sequencing data (Gb) from the topping treatment (TT) and control (CK) experiments

Sequencing methods	Control (CK)				Topping (TT)			
	Rep1	Rep2	Rep3	Total	Rep1	Rep2	Rep3	Total
RNA-Seq	17.74	16.79	16.02	50.55	17.71	17.37	17.03	52.11
circRNA-seq	20.30	22.44	25.52	68.26	24.61	29.24	26.75	80.6
miRNA-Seq	4.10	3.60	4.60	12.30	5.30	4.50	3.00	12.80
ssRNA-Seq	12.18	12.12	11.98	36.28	11.14	12.11	11.93	35.18

**Table 2:** Number of coding and three types of non-coding RNAs identified in tobacco

Type	Loci	Transcripts	Identified by
mRNA	85,570	145,503	Sierro et al., 2014 (79,821 expressed in this study)
lncRNA	7423	8520	Only this study
miRNA*	981	981	This study, Li et al., 2015 and miRBase V22
circRNA	12,414	13,957	Only this study

* 688 miRNAs were identified in this study

### Expression profiling of coding RNAs and three types of ncRNAs in tobacco roots

**Fig. 1:**
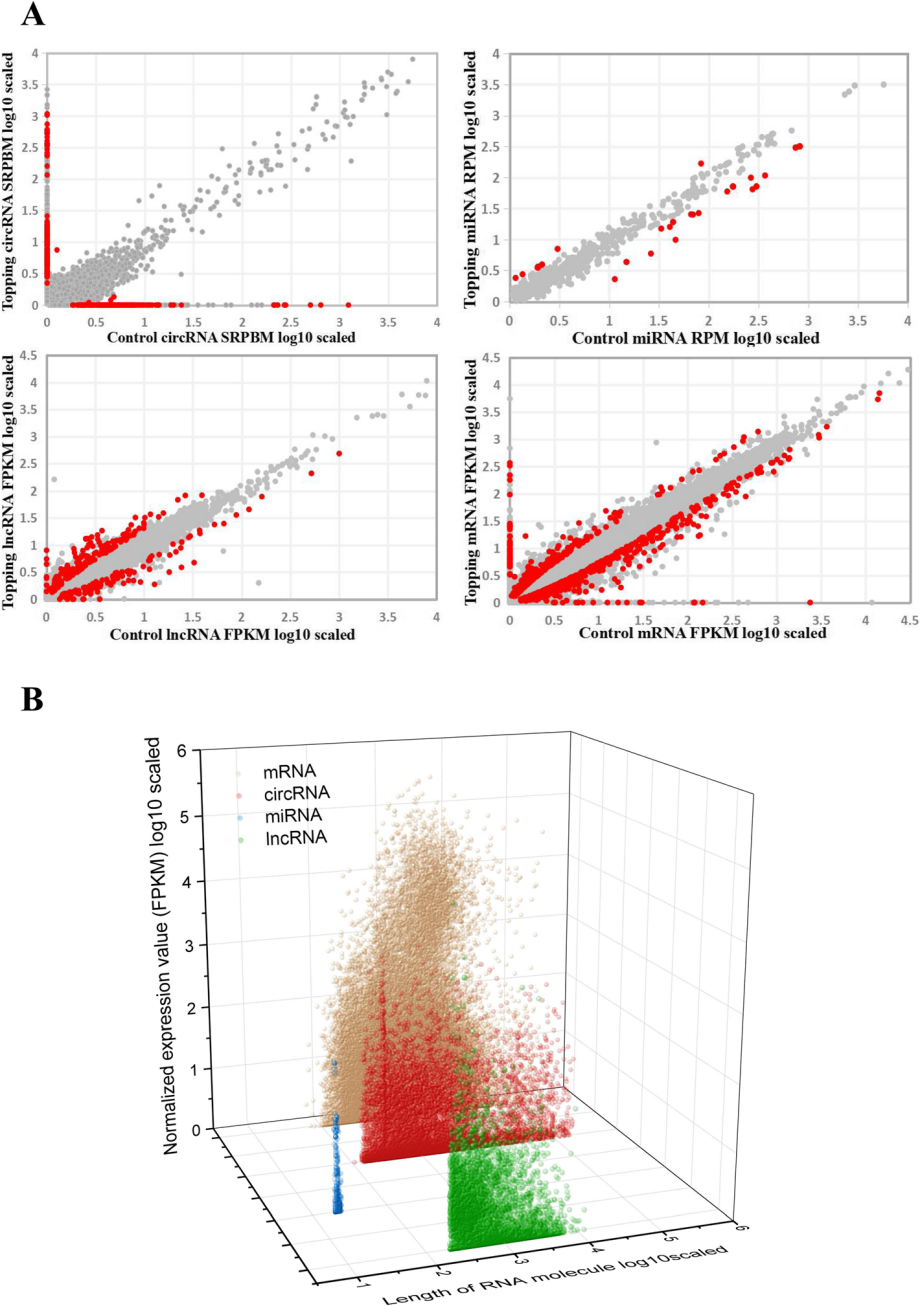
Expression profiling of mRNAs, circRNAs, miRNAs and lncRNAs in tobacco. **a** Normalized expression values of mRNAs (FPKM), circRNAs (SRPBM), miRNAs (RPM) and lncRNAs (FPKM) in control vs. topping-treated. Red and gray points represent significantly differentially expressed RNAs and not differentially expressed RNAs, respectively; **b** Normalized expression values (FPKM) of different length of mRNAs, circRNAs, miRNAs and lncRNAs in tobacco after topping treatment

**Fig. 2:**
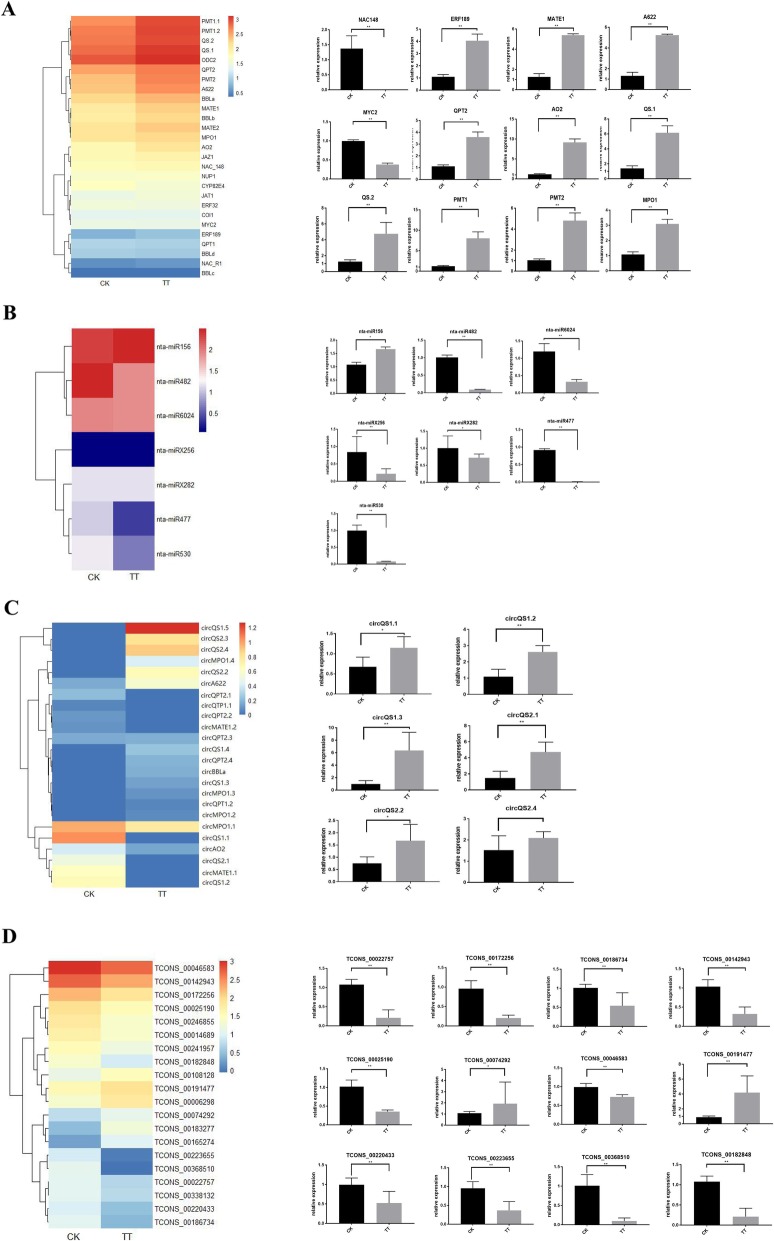
Normalized expression levels of 4 types of topping responsive RNA molecules and verified by qRT-PCR. **a**) Normalized expression level of 27 nicotine biosynthesis and metabolism related mRNAs based on RNAseq data and partial RT-qPCR results. **b** Normalized expression level of 7 topping response miRNAs on RNAseq data and partial RT-qPCR results. **c** 24 circRNAs generated from nicotine biosynthesis and metabolism related genes on RNAseq data and partial RT-qPCR results. **d** Normalized expression level of 20 topping response lncRNAs based on RNAseq data and partial RT-qPCR results. Data are means ± SD (*n* = 3) *P < 0.05, **P < 0.01 (One-way ANOVA followed by LDS test)

Expression levels of mRNA and lncRNA were quantified using FPKM, while SRPBM and RPM were used to determine the expression levels of circRNA and miRNA, respectively. In the topping-treated sample, 1058 significantly differentially expressed genes (DEGs) (|log2 (fold-changes)| ≥1, FDR < 0.05) were identified. As expected, most genes, such as *QPT2*, *QS* and *PMT2,* of the nicotine biosynthesis pathway were significantly up-regulated in response to topping. For ncRNAs, 315 (159 up-regulated and 156 down-regulated), 41 (15 up-regulated and 26 down-regulated) and 295 (178 up-regulated and 117 down-regulated) differentially expressed circRNAs, miRNAs and lncRNAs were identified, respectively (Fig. 1a). Nine mRNAs involved in nicotine biosynthesis and 10 mRNAs related to nicotine degradation, transportation, or topping responsive TFs were selected for qRT-PCR validation (Fig. 2a). All genes related to nicotine biosynthesis (*QPT2*, *QS*, *PMT2*) or transportation (*MATE1*, *MATE2*, *A622*) were confirmed to be significantly up-regulated, while topping responsive TFs like *NAC148* and *MYC2* were confirmed to be significantly down-regulated (Fig. 2a). Besides, the differential expression patterns of 6 of the 7 selected miRNAs, including 3 targeting nicotine biosynthesis genes, were also verified by qRT-PCR (Fig. 2b). qRT-PCR verification was also conducted for 12 lncRNAs and 17 circRNAs, including 15 circRNAs generated from nicotine biosynthesis genes. The expression changes in responsive to the topping-treatment were verified for 2 up-regulated and 10 down-regulated lncRNAs (Fig. 2d). For the circRNAs, the topping-induced expression patterns were verified for 9 out of the 17 selected ones, just as their topping-induced parental genes (Fig. 2c). Several down-regulated circRNAs generated from *MATE1* and *QS* according to the circRNA-seq data failed to be detected.

In order to effectively compare the expression level of the four types of RNAs, a unified normalization standard is required. The expression level of circRNA and miRNA was converted into FPKM and plotted FPKM of the four types of RNAs based on their length (Fig. 1b). The mean expression levels of mRNAs, circRNAs, miRNAs and lncRNAs were 252.38, 7.51, 4.00, and 15.11, respectively, suggesting that ncRNAs were expressed at much lower levels than protein coding genes in tobacco roots. However, of all the RNAs detected in this study, both the most significantly down-regulated (log2|FC| = − 18.8508) and up-regulated (log2|FC| = 19.324) transcripts in the topping-treated sample were lncRNAs. The transcript length of the expressed RNAs was also compared. The most highly expressed circRNAs have a length of 300 bp - 2000 bp even though the average circRNA length was around 5 kb, while the most highly expressed mRNAs and lncRNAs have a length of 2000 bp - 8000 bp and 200 bp - 1000 bp, respectively.

### Co-expression of the four types of RNA molecules responsive to topping in tobacco roots

Most ncRNAs have not been annotated and an unknown function. To identify potential roles of the ncRNAs, a co-expression network for the four types of RNA molecules was constructed by calculating Pearson’s correlation coefficient value (the absolute value of PCC > 0.99, *p*-value < 0.05 and FDR < 0.05). From the network, we could see that coding and noncoding RNA molecules may affect plant defensive mechanism in various ways by interacting with each other.

**Fig. 3:**
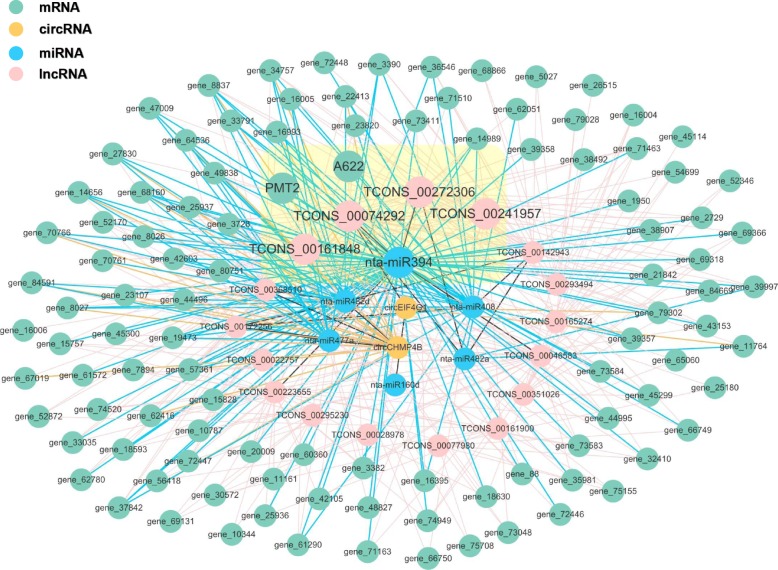
Co-expression network of differentially expressed mRNAs, circRNAs, miRNAs and lncRNAs. The network was established based on Pearson correlation coefficient (the absolute value of PCC > 0.99, *p*-value < 0.05 and FDR < 0.05). The blue, orange and pink lines represented the co-expression relationship of mRNA-miRNA, mRNA-circRNA and mRNA-lncRNA respectively. The yellow block in the middle of the network indicated the RNAs involved in nicotine biosynthesis

As shown in Fig. 3, after applying stringent filtering steps 6 conserved miRNAs were found in the co-expression network. Two miRNAs (miR482a and miR482d) of the 6 miRNAs identified belong to the miR482 family. miR482a/d had co-expression patterns with 27 mRNAs, including several targeting *NBS* genes as previously reported [30]. miR482a/d, miR394, miR408 and miR477a were co-expressed not only with mRNAs but also with lncRNAs. Both miR482d and miR477a also had a positive correlation with circCHMP4B. miR160d showed only negative correlation with circEIF4G1 generated from *EIF4G1*. circCHMP4B and circEIF4G1 were the only two circRNAs included in the network, since most co-expressed pairs between circRNA and other types of RNA molecules were not able to be connected to a third type of RNA. However, a larger number of lncRNAs (18) was presented in the network and showed a co-expression pattern with 106 mRNAs as well as the aforementioned 6 miRNAs and 2 circRNAs. With the network, it was not only possible to assess the function of ncRNAs by means of well-studied and co-expressed protein-coding mRNAs, but also to deduce the ncRNAs related to mRNAs of interest.

Closer examination of the genes of the nicotine biosynthesis pathway showed that *A622* and *PMT* were present in this co-expression network and that miR394 showed a negative correlation with these two genes. *PMT* also had a positive co-expression relationship with lncRNA TCONS_00161848 and a negative co-expression relationship with lncRNAs TCONS_00241957 and TCONS_00272306. *A622* was also negatively co-expressed with TCONS_00272306, although it had a positive co-expression relationship with another lncRNA (TCONS_00074292). circCHMP4B and circEIF4G1 were present in the network, but their parental genes (*CHMP4B* and *EIF4G1*) were not related to nicotine metabolism, thus no putative circRNA-containing network related to nicotine biosynthesis was identified in this study. Nevertheless, it was possible to find 5 nicotine biosynthesis related co-expression networks containing miRNA, mRNA and lncRNA (i.e.*,* miR394-A622-TCONS_00272306, miR394-PMT2-TCONS_00272306, miR394-A622-TCONS_00074292, miR394-PMT2-TCONS_00241957 and miR394-PMT2-TCONS_00161848).

### miRNA regulation network

To further provide convincing evidence for the crosstalk among miRNA, lncRNA, circRNAs and mRNA, the differential expression (|log2FC| ≥ 1) pattern of the RNAs involved and their physical interaction potential were checked.

Based on target prediction, 678 mRNAs were predicted potentially regulated by 114 miRNAs, including the miRX27-*QPT2* pair studied in our previous work [22]. Many miRNA-targeted mRNAs were reported to be stress associated. The most significantly down-regulated miRNA identified in this study was miR530, which targets *KIP1* that was up-regulated after the topping treatment. Upon the topping treatment, miRX393 was significantly up-regulated while its target *CYP450* was down-regulated. Many circRNAs were predicted to having interactions with miRNAs. Linear regression analysis indicated that miRNAs had negative expression correlation with their predicted target genes (R^2^ = 0.52, Additional file 4: Figure S4A) and target circRNAs (R^2^ = 0.72, Additional file 4: Figure S4B). Interestingly, most miRNAs (88.2%) targeting circRNAs were up-regulated after the topping treatment. Majority of these miRNAs are conserved ones, for example, 40 of the 114 miRNAs involved in the interaction between miRNA and mRNA, and 23 of the 36 miRNAs involved in the interaction between miRNA and circRNA were conserved. Some well-known miRNA families like miR156, miR482 and miR477 were found in both interactions, particularly miR156, more than 10 members were found to be involved in the two interactions. As for lncRNA-miRNA interaction, differentially-expressed miRNA and lncRNA that was predicted to be eTM of the miRNA were analyzed, and no statistical relationship could be concluded. The relationship between circRNAs and their parental genes was analyzed using the genes generating a single circRNA isoform. Forty-nine (49) circRNA-mRNA pairs with a highly positive co-expression relationship were found (R^2^ = 0.34, Additional file 4: Figure S4C). The most significantly differential expressed circRNA was circLASPO, which was co-upregulated after the topping treatment with its parental gene LASPO (L-aspartate oxidase), a gene related to the production of aspartate.

### GO and KEGG analysis of parental genes generating differentially expressed circRNAs

**Fig. 4:**
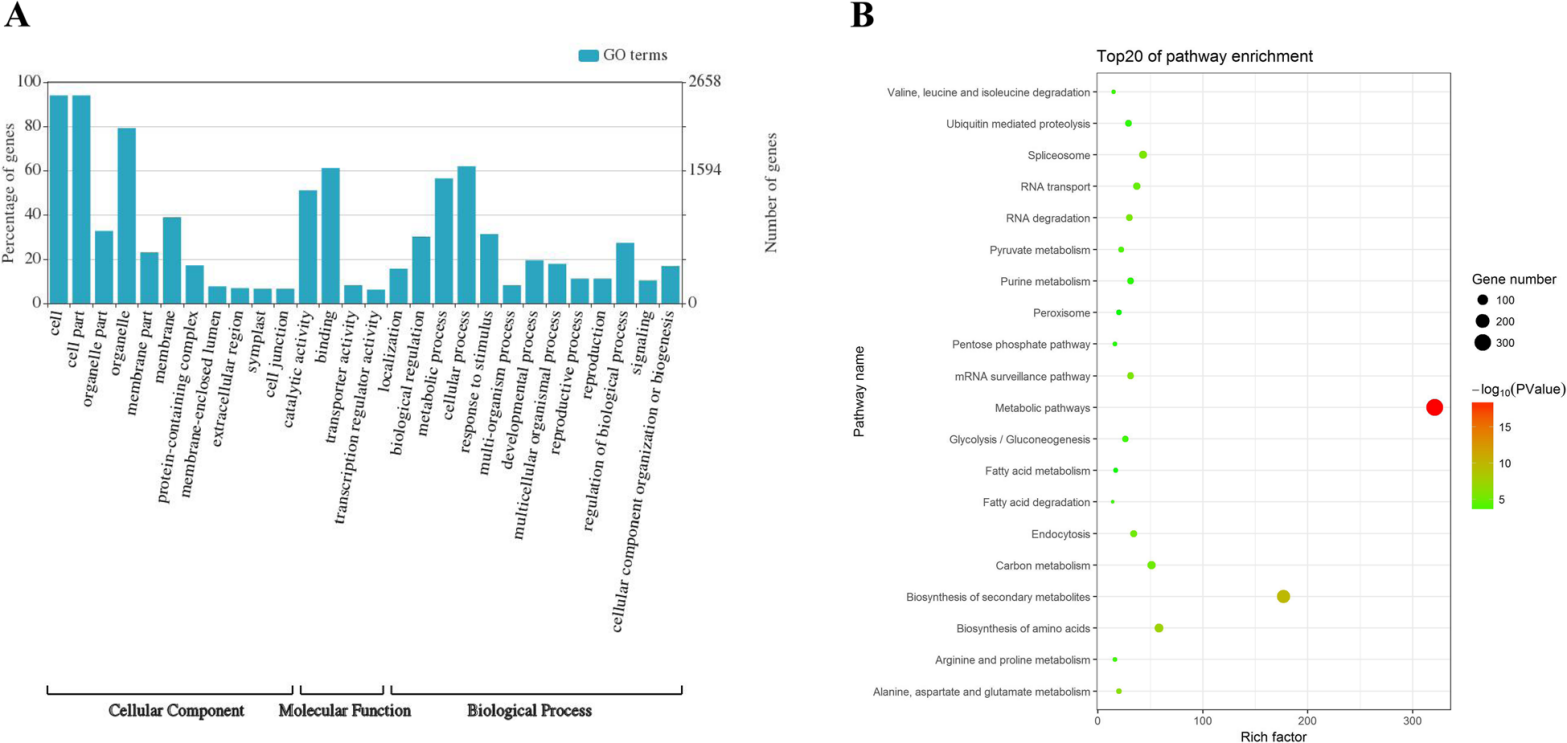
Functional annotations of protein coding parental genes generating differentially expressed circRNAs. **a** Gene ontology (GO) classification of parental genes of circRNAs; **b** The top 20 enriched KEGG pathways of circRNA-producing parental genes

The composition and potential function of circRNAs have not been previously reported in tobacco. Here GO and Kyoto Encyclopedia of Genes and Genomes (KEGG) pathway analyses for the parental genes of differentially expressed circRNAs (|log2FC| ≥ 1) were carried out to understand the topping responsive pathways potentially regulated by circRNAs. Three GO categories were assigned to the 5147 parental genes of 7235 circRNAs (Fig. 4a). In the biological process category, “metabolic process”, “cellular process” and “regulation of biological process” were among those significant GO subcategories. Among them, GO: 0050896 (Response to stimulus) was significantly enriched for 673 mRNAs. According to KEGG analysis, the most highly represented pathways were those related to biosynthesis of secondary metabolites (Fig. 4b), implying that many parental genes of the differentially expressed circRNAs in the topping-treated tobacco roots are involved in synthesis of secondary metabolites, including nicotine.

### The regulation network of the three types of RNA molecules involved in the nicotine biosynthesis pathway

Based on BLASTp search using known genes [31], 27 tobacco genes related to nicotine biosynthesis and metabolism were identified (Fig. 2a). From 10 of these genes, 24 exonic-circRNAs and one intronic circRNA were identified (Fig. 2c). Some genes generate multiple circRNAs, such as *QS.1* producing 5, and *QPT2*, *MPO1* and *QS.2* each producing 4. For the nicotine biosynthesis related miRNAs, in addition to those identified in our previous study [22], one conserved (miR6024) and 6 novel miRNAs were newly found to target 5 of the 27 genes related to nicotine biosynthesis (Fig. 2b). As the interaction principle between lncRNAs and other types of RNA molecules is still under debate and, thus, no prediction attempt was made, this study therefore focused on the construction of miRNA-circRNA-mRNA networks involved in regulation of nicotine biosynthesis.

**Fig. 5:**
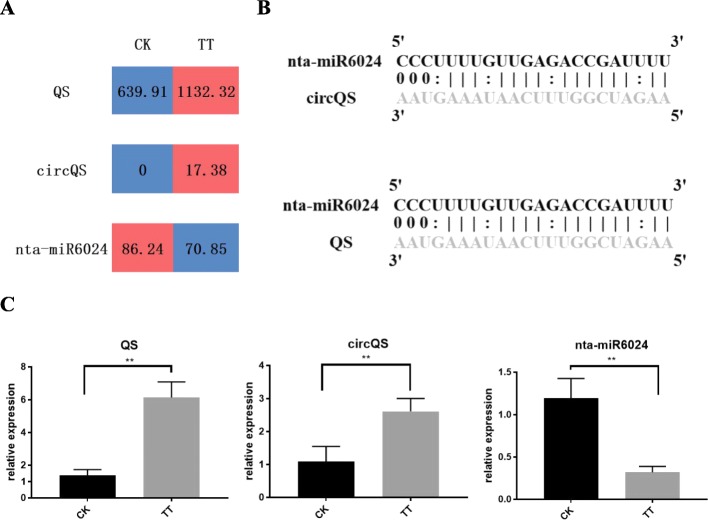
The putative regulation network of non-coding RNAs involved in *QS* gene in nicotine pathway. **a** Normalized expression levels of *QS*, circQS and nta-miR6024 based on the RNA sequencing data; **b** Alignment of nta-miR6024 and its target RNAs. Base pairing between miRNA and its targets is shown, in which a vertical line means a Watson-Crick pair, two dots represent a G-U pair, and 0 means a mismatch; **c** qRT-PCR analyses of *QS*, circQS and nta-miR6024 in roots from the topping-treated and control plants

**Fig. 6:**
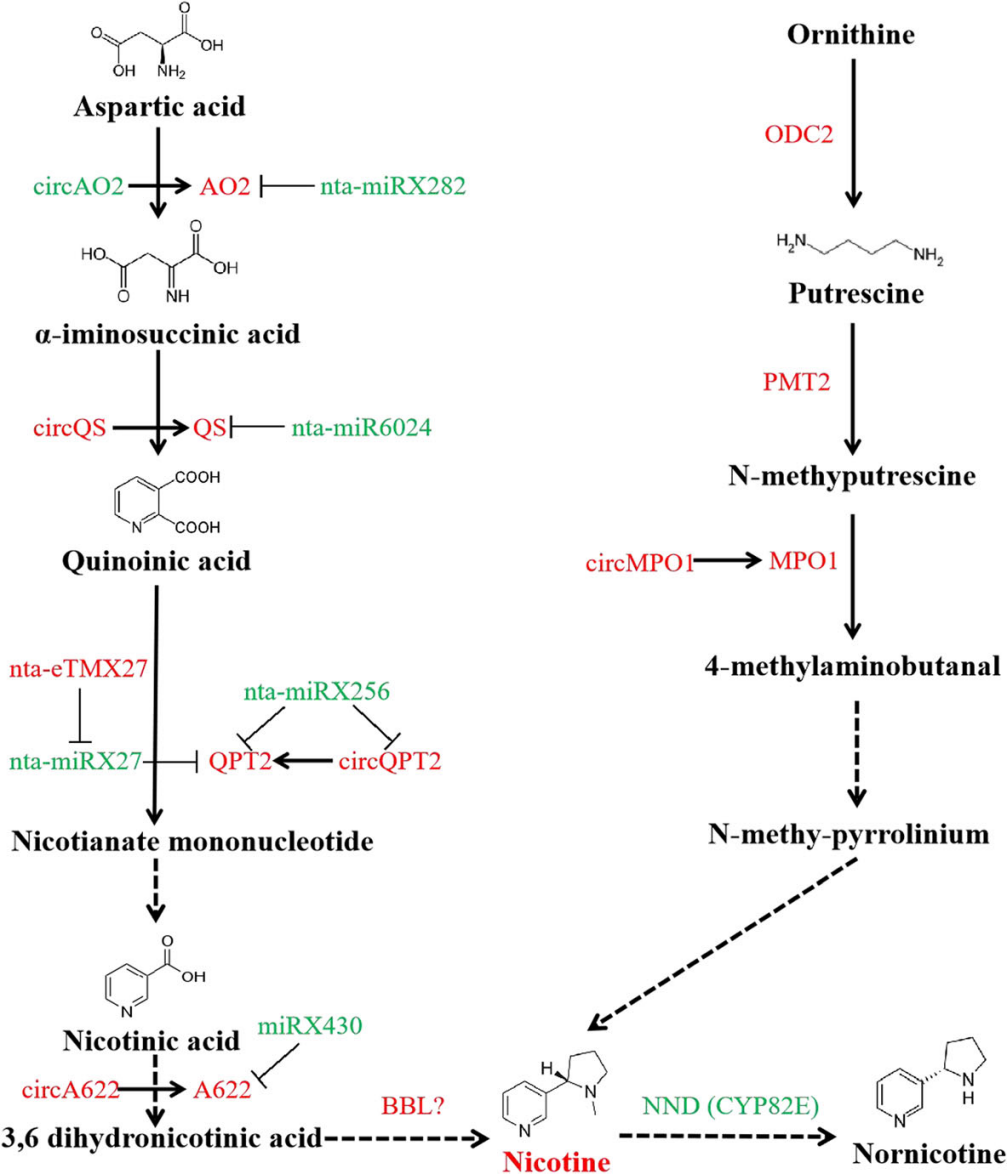
The potential interaction of RNA molecules involved in the nicotine biosynthesis pathway in tobacco root. The differential expressed circRNAs which generated from parental genes involved in nicotine biosynthesis pathway were presented. The differential expressed miRNAs which target to genes or circRNAs were also shown in the figure. The nicotine biosynthesis pathway diagram drew based on previous studies in tobacco. The up-regulated genes/non-coding RNAs after topping treatment were presented in red, while down-regulated ones were in green

Fourteen (14) out of the 27 genes related to nicotine biosynthesis were significantly up-regulated after the topping treatment (Fig. 2a). From 8 of those gene significantly up-regulated, 16 differentially expressed circRNAs (|log2FC| ≥ 1, adjusted *P*-value≤0.05) were identified based on analysis of circRNA-seq data. The differential expression pattern was verified by qRT-PCR for 9 of the 16 circRNAs (generated from 4 parental genes). Based on the expression analysis and identification of the interaction sites, three networks involving mRNA, miRNA and circRNA were uncovered. *QS*, that contributes to both the nicotine and NAD pathways, was involved in the network QS-circQS-miR6024 (Fig. 5). The target sites of miR6024 were identified in both *QS* and circQS, which were significantly up-regulated while their repressor miR6024 was down-regulated (Fig. 5c). Due to *QS* and circQS having identical target sites of miR6024, their repression by miR6024 would be competitive. Of the components of the network AO2-circAO2-miRX282, *AO2* gene was significantly up-regulated after the topping treatment, while circAO2 and miRX282 were slightly down-regulated (Fig. 2b, Fig. 2c). In the third network QPT2-circQPT2-miRX256, the miRNA did not target circQPT2 but *QPT2*. The expression level of *QPT2* was significantly up-regulated after the topping treatment while that of miRX256 was quite low and down-regulated (Fig. 2b). Multiple circRNAs were identified in *QPT2.* Two (circQPT2.1 and circQPT2.2) were only identified in the control while the third circRNA (circQPT2.3) was only found in the topping treatment. All three circRNAs had a low expression level. Of the three networks, QS-circQS-miR6024 showed the highest expression levels of its components (Fig. 5c and Fig. 2), implying its importance in regulating nicotine biosynthesis. Conclusively, as shown in Fig. 6, coding genes were not the only determinant in nicotine biosynthesis in tobacco root, the interaction with non-coding RNAs, such as circRNAs and miRNAs, also extensively involved in it. To understand the regulation mechanism of non-coding RNAs involved in nicotine biosynthesis, further efforts are still needed.

## Discussion

Crosstalk and functional activities of ncRNAs and coding RNAs in plants are organized in complex modes. Interaction studies so far in plants mostly focused on two types of RNAs, such as miRNAs and their mRNA targets [1], circRNAs and their parental genes [32], and miRNAs and their eTM lncRNAs [2]. Regarding regulation of nicotine biosynthesis and other secondary metabolism in tobacco, a role of ncRNAs has rarely been demonstrated [33]. The present work investigated the dynamic changes of mRNAs, miRNAs, circRNAs and lncRNAs, and their interactions in response to topping treatment in tobacco, with a focus on coding and non-coding RNAs related to nicotine biosynthesis. Our results provide evidence for the involvement of ncRNAs and their interactions with coding genes in nicotine biosynthesis and the foundation for further functional characterization of the networks involving both coding and non-coding RNAs in tobacco.

The most abundant transcripts in tobacco were coding RNAs. Among the three types of ncRNAs, circRNA was the predominant one probably due to the high frequency of alternative back splicing of linier genes in tobacco [34], and miRNA was the least abundant one. A large number of tobacco circRNAs were identified in this work, but *A. thaliana* or *O. sativa* have approximately 3 times more circRNAs than tobacco, even though tobacco has a much larger genome size and encodes many more genes (Additional file 5: Table S1). This difference could partially be the result of the many different bioinformatics tools having been used in circRNA identification in *A. thaliana* or *O. sativa* [29]. As the limitation of the circRNA enrichment technology and no single method dominated on all of the metrics such as precision and sensitivity to date, use of different bioinformatic tools would compensate the drawbacks of each individual tool to get comprehensive results. Some lowly expressed circRNAs failed to be constantly identified also lead to false negative results. A total of 688 miRNAs were identified in this study, including 268 conserved ones from 38 families and 420 novel miRNAs from 393 families. A significant portion of these miRNAs were newly identified in this study, which provided an important complementation for the previous reported ones [17–19, 22]. miRNAs are usually much more conserved than other ncRNAs. Nearly 39% of the miRNAs identified in this study were conserved ones (Additional file 1: Figure S1A). The abundance of lncRNAs identified in tobacco roots were also investigated and compared with the lncRNA number in *A. thaliana* and *O. sativa* [35, 36]. It showed a positive correlation between the abundance of lncRNAs and the complexity of plant genome.

This study, for the first time in plants, constructed a lncRNA-miRNA-circRNA-mRNA co-expression network based on differentially expressed RNAs with stringent filters (the absolute value of PCC > 0.99, *p*-value < 0.05 and FDR < 0.05). Interestingly, even though circRNAs were the predominant ncRNAs, they were the least ones with a co-expression relationship with mRNAs, instead lncRNA-mRNA was the most favorable co-expression pair (Fig. 3). The phenomenon can be explained that a large amount of circRNAs was only identified specifically in untreated or topping group. Most miRNAs (88.2%) targeting circRNAs were up-regulated after the topping treatment, and consequently the expression levels of all the targeted circRNAs were decreased. This hinted us that miRNAs could shift the balance between mRNA and circRNA towards mRNA to activate the cell self-protection. Nevertheless, most of the significantly differentially expressed circRNAs exhibited an expression profile similar to their parental genes (Additional file 4: Figure S4C), suggesting a role of plant circRNAs in regulation of their parental genes [32]. Only two circRNAs represented in the co-expression network, largely due to the low expression level of circRNAs and their unique to one of the two samples used. This feature of circRNAs is quite different from that of other two types of ncRNAs, because 5 (miR394-A622-TCONS_00272306, miR394-PMT2-TCONS_00272306, miR394-A622-TCONS_00074292, miR394-PMT2-TCONS_00241957 and miR394-PMT2-TCONS_00161848) co-expressed miRNA-mRNA-lncRNA modules were found. The conserved miR394 targeting F-box genes [37] was the only RNA molecule involved in all the 5 modules. Both *A662* and *PMT2* are not target genes of miR394, suggesting that co-expression of miR394 with *A662* or *PMT2* could be achieved through the lncRNAs of the modules. The majority nodes of the co-expression network were mRNAs, ncRNAs occupied only a small number of nodes of the co-expression network, suggesting that ncRNAs-mediated regulation of cellular processes might be mainly achieved by interacting with mRNAs rather than with other ncRNA molecules.

Co-expression analysis uncovered the topping-responsive profile of ncRNAs and coding RNAs, interaction analysis further provided us the knowledge about the RNA crosstalk in tobacco roots. Among the miRNA-mRNA interaction pairs, 41 miRNAs were significantly differentially expressed in response to the topping treatment. Most (31) of these 41 were conserved miRNAs, including those that have been reported to be topping or wounding responsive, such as miR160, miR164, miR394, miR408, miR477 and miR1919 [18, 22]. For instance, miR164 and its target *NtNAC-R1* was down- and up-regulated in response to topping, respectively, consistent with the results reported previously [21]. The target genes of miR160, miR394, miR408, miR477 and miR482 generated large amount circRNAs, but only circRNAs from *CHMP4B* and *EIF4G1* were found to form the networks with three of these miRNAs (i.e., CHMP4B*-*miR477/482-circCHMP4B and EIF4G1*-*miR160-circEIF4G1).

Although *CHMP4B* and *EIF4G1* are not nicotine biosynthesis related genes, it was possible to find 3 networks involving mRNAs, circRNAs and miRNAs with a potential regulatory role in nicotine biosynthesis. In two of these networks, QS-circQS-miR6024 and AO2-circAO2-miRX282, miR6024 and miRX282 have target sites on both mRNA (*QS* and *AO2,* respectively) and circRNA (circQS and circAO2, respectively). Because each miRNA has the identical targets in the circRNA and its parental gene, binding of the miRNA to each of its two targets would be competitive. In view of the negative correlation between miRNA and its targets, it is expected that binding of the miRNA to circRNA would relax the miRNA-mediated cleavage of its target mRNA; therefore, the role of the circRNA is similar to that of eTM, i.e. function as the sponge of miRNAs but probably in a cleavage manner. This relationship among miRNA and its target mRNA and circRNA from the target mRNA suggests that the expression of *QS* and *AO2*, consequently nicotine biosynthesis, is under fine regulation mediated by miRNAs.

In summary, our comprehensive analyses of mRNAs and ncRNAs (miRNAs, lncRNAs, circRNAs) in tobacco roots and identification of coding and non-coding RNAs and their interaction networks in responsive to topping treatment enabled us to draw an overall picture of the transcriptome profile of all the four types of RNA molecules and offered us an insight into the crosstalk amongst these RNAs in tobacco.

## Methods

### Plant materials and treatment

All samples were collected from tobacco (*Nicotiana tabacum*) ‘K326’ root. Tobacco plants were grown at 25 °C and 65% humidity in a growth chamber to reduce external environmental effects on the biosynthesis of nicotine alkaloids with a day-night cycle of 16 h light and 8 h darkness. At least three 50-d-old (days after seeding) plants were used for topping treatment (i.e., removal of the floral head and the upper young leaves of the tobacco plants), and plants of a similar size without topping treatment were used as control. After topping treatment, the plants were kept for another 48 h in the growth chamber before sample collection.

### Library preparation and Illumina sequencing

Total RNAs were isolated from root samples of topping-treated and untreated plants (each treatment with three biological replicates) using MiniBEST Plant RNA Extraction kit (TAKARA) following the manufacturer’s procedure. Total RNA concentration and purity were assessed by Nanodrop ND 2000 (Thermo Fisher Scientific).

For mRNA sequencing library preparation, mRNA was enriched and purified with oligo (dT)-rich magnetic beads and then broken into short fragments. Six libraries (three libraries each for the topping-treated and untreated samples, same for miRNA-seq, circRNA-seq and lncRNA-seq) were generated and sequenced using an Illumina HiSeqTM 2500 platform (BIOMARKER, China). miRNA libraries were prepared by TruSeq Small RNA Sample Prep Kits (Illumina) and sequenced in a single-end (50 bp) manner on an Illumina Hiseq 2500 platform following the vendor’s recommended protocol. LncRNA and circRNA libraries were created using the mRNA-Seq sample preparation kit (Illumina) after depletion of ribosomal RNAs by the Ribo-Zero Gold Kit (Illumina). Paired-end sequencing was then performed on an Illumina Hiseq 4000 and Illumina Hiseq X for lncRNA-seq and circRNA-seq, respectively.

### Computational identification of circRNAs

The adaptors and low-quality reads were removed from the sequencing raw data using Trimmomatic v 0.36 [38]. Filtered clean reads were mapped to the *N. tabacum* reference genome (Sierro et al., 2014) using Bowtie2 v 2.2.6 [39], TopHat v 2.1.1 [40] and STAR v 2.5.2 [41] to get different format of mapping results. All the unmapped reads were then used as input files to CIRI v 2.0.6 [42], find_circ v 1.0 [43] and PcircRNA_finder [44] for back-spliced junction and circRNAs identification.

### Prediction of miRNAs in tobacco

The clean reads were first compared with the annotated non-coding RNA sequences, including plant snoRNA (version 1.2; http://bioinf.scri.sari.ac.uk/cgibin/plant_snorna/), tRNA (http://gtrnadb.ucsc.edu/), rRNA (http://rfam.xfam.org/) and rasiRNA (http://www.girinst.org/) to remove those matching to these known non-coding RNAs.

The remaining clean reads were then aligned to the *N. tabacum* genome (Sierro et al., 2014) for identification of miRNAs. Mireap (http://sourceforge.net/projects/mireap/) was applied to predict secondary hairpin structures of MIRNAs with the following parameters: the hairpin structure has a free energy lower than − 18 kcal mol^− 1^; the space between miRNA and miRNA* is less than 300 nt; miRNA and miRNA* have at least 16 matched nucleotides and fewer than four unmatched nucleotides. Only small RNAs with at least two reads in a library were used for miRNA prediction. A miRNA was considered as conserved if its mature sequence had ≤2 mismatches with the miRNAs deposited in the miRBase (http://www.mirbase.org, release 21), otherwise the miRNA was considered as a novel miRNA [45].

### Computational identification of lncRNAs

All the raw reads from transcriptome sequencing were treated using Trimmomatic v 0.36 [38] with the default parameters for quality control. The clean data were then mapped to the *N. tabacum* genome using Tophat v 2.1.1 [40]. For each mapping result, Cufflinks v 2.1.1 [46] was used in transcript assembly. For strand-specific RNA-seq datasets, the parameter “--library-type fr-firststrand” was employed. All transcriptomes were then merged based on the annotation file of the reference genome to generate a final transcriptome using Cuffmerge. Cuffdiff was used to estimate the abundance of all transcripts based on the finally merged transcriptome. We then used the following six filters to shortlist the bona fide lncRNAs from the obtained final transcriptome assembly: (1) transcripts without strand information were removed; (2) all single-exon transcripts that are within a 500-bp flanking region of known transcripts and in the same direction as the known transcripts were discarded; (3) transcripts overlapped with mRNAs annotated in the reference genome were deleted; (4) transcripts with FPKM scores < 0.5 (2 for single-exon transcripts) and shorter than 200 bp were discarded; (5) the coding potential value of each transcript was calculated using CPC and those with CPC scores > 0 were discarded; (6) the remaining transcripts were searched against the Pfam database by HMMER to remove transcripts containing known protein domain. The remaining transcripts were regarded as expressed candidate lncRNAs.

### Expression analysis and mRNA-circRNA-miRNA-lncRNA interaction network construction

Quantifications of the expression level of genes, circRNAs and miRNAs were performed using FPKM (fragments per kilobase of transcripts per million mapped reads), SRPBM (number of circular reads/number of mapped reads (units in billion)/read length), and RPM (reads count of miRNAs/clean reads× 10^6^), respectively. Differential expression of genes, circRNAs and miRNAs was profiled with the Rpackage edgeR. *P*-values were adjusted using the Benjamini and Hochberg’s approach. Differentially expressed mRNAs, circRNAs, miRNAs and lncRNAs were defined by different standard in terms of the adjusted P-value and the absolute log2 (fold-changes) as shown in Additional file 8: Table S4, Additional file 9: Table S5, Additional file 10: Table S6, Additional file 11: Table S7. To compare the expression level of the four types of RNA molecules in a unified manner, they were normalized as FPKM_mRNA_ (number of mRNA reads/number of mapped reads (units in billion)/mRNA transcript length), FPKM_circRNA_ (number of circRNA reads/number of mapped reads (units in billion)/circRNA junction length), FPKM_miRNA_ (number of miRNA reads/number of mapped reads (units in billion)/miRNA transcript length), FPKM_lncRNA_ (number of lncRNA reads/number of mapped reads (units in billion)/lncRNA transcript length). Pearson’s correlation coefficient was used to measure the strength of the linear dependence of two variables. Pearson’s correlation value was calculated for the mRNA-circRNA-miRNA-lncRNA co-expression network. The networks with a Pairs’ absolute coefficient value over 0.8 and a P-value below 0.05 were selected. The network results were visualized using Cytoscape v 3.6.0 to show the co-expression relationships [47]. Targets of miRNAs were predicted via the online sever psRNATarget with the default settings [48].

### Annotation of nicotine biosynthesis and catabolism related genes

The clean reads of RNA-Seq data were mapped to the same *N. tabacum* reference genome mentioned above using hisat2 v 2.1.0 [49]. The annotation of nicotine-related genes in *N. tabacum* was carried out using the method described previously by Shen et al. (2015) with minor modifications, i.e. an E-value <1e-5 and sequence identify > 50% were used in BLASTp search of homologous genes related to nicotine biosynthesis and catabolism [50].

### Expression validation of mRNAs and ncRNAs in tobacco

qRT-PCR was used to validate the expression levels of selected mRNAs, miRNAs, circRNAs and lncRNAs. Total RNAs isolated from roots of topping-treated or untreated plants were used in the first-strand cDNA synthesis using the HiScript II 1st Strand cDNA synthesis kit (Vazyme) according to the manufacturer’s instruction. The Oligo (dT) and random hexamers supplied in the kit were used in generation of the 1st strand cDNA for quantification of mRNAs and lncRNAs, respectively. The 1st strand cDNAs generated using random hexamers were also used in analysis of circRNAs. To validate circRNAs identified in this study, polymerase chain reactions (PCRs) were performed using a set of divergent primers and a set of convergent primers that were used as a control. For miRNA quantification, a stem loop primer was used in reverse transcription. qPCR was performed with the ChamQ SYBR qPCR Master Mix kit (Vazyme) on the LightCycler 96 platform (Roche). The PCR procedure for circRNAs, lncRNAs and target genes was as following: 95 °C for 30 s, 40 cycles at 95 °C for 10 s, 60 °C for 30 s and then 72 °C for 30 s. The PCR procedure for miRNAs was as following: 95 °C for 5 min, 40 cycles at 95 °C for 10 s, 60 °C for 30 s and then 72 °C for 20 s. In both programs, the melting curve and amplification curve were examined to evaluate the specificity of amplification. The relative expression levels were analyzed by the 2 − ΔΔct method. *U6* was used as the internal control for miRNAs. *EF-1α* was used as the internal control for circRNAs, lncRNAs and target genes. All the qRT-PCR reactions were assayed in triplicates and the primers used in our study were listed in Additional file (Additional file 6: Table S2, Additional file 7: Table S3).

## Conclusions

In this study, 688 miRNAs, 7423 lncRNAs and 12,414 circRNAs were identified in tobacco roots. Although not all four types of RNAs, several networks involving three types of RNAs (e.g. mRNA-miRNA-circRNA) related to nicotine biosynthesis were uncovered. Our results illustrated the transcriptomic profiles of tobacco roots, an organ responsible for nicotine biosynthesis, suggesting that mRNAs always play the most important roles, while ncRNAs are also expressed extensively for topping treatment response, especially circRNAs are the most activated in the ncRNA pool. NcRNAs appear to form interaction complex or network for the regulation of stress response such as nicotine biosynthesis. Taken together, the study enhanced our understanding of the RNA landscape in tobacco and will facilitate functional characterization of mRNAs and ncRNAs in biotic/abiotic responses in tobacco.

## Supplementary information

**Additional file 1: Figure S1.** Genome-wide identification of miRNAs in tobacco.

**Additional file 2: Figure S2.** Genome-wide identification of lncRNAs in tobacco.

**Additional file 3: Figure S3.** Features of circRNAs identified in tobacco and compared with other plants.

**Additional file 4: Figure S4.** Co-expression patterns of coding RNAs and three types of non-coding RNAs.

**Additional file 5: Table S1.** Number of coding and three types of non-coding RNAs identified in other plants.

**Additional file 6: Table S2.** Primers used in qRT-PCR for miRNA.

**Additional file 7: Table S3.** Primers used in qRT-PCR for mRNA, lncRNA and circRNA.

**Additional file 8: Table S4.** Summary of expression changes of circRNAs between topping treatment (TT) and control (CK) group.

**Additional file 9: Table S5.** Summary of expression changes of miRNAs between topping treatment (TT) and control (CK) group.

**Additional file 10: Table S6.** Summary of expression changes of lncRNAs between topping treatment (TT) and control (CK) group.

**Additional file 11: Table S7.** Summary of expression changes of mRNAs between topping treatment (TT) and control (CK) group.

## Data Availability

All the sequencing data generated in this study was submitted to NCBI with accession number PRJNA526387.

## Abbreviations

eTM: endogenous target mimicry
ncRNA: non-coding RNA
PMT: Putrescine methyltransferase
QPT: quinolinic acid phosphoribosyl transferase
QS: encoding quinolinate synthase
TF: Transcription factor
