# Supplementary material for: A transcriptomic profile of topping responsive non-coding RNAs in tobacco roots (Nicotiana tabacum)
Source: BMC Genomics. 2019 Nov 14;20:856. doi: 10.1186/s12864-019-6236-6 (PMC6854694; doi:10.1186/s12864-019-6236-6)
Supplement: Supplementary file 1 — Additional file 1: Figure S1. Genome-wide identification of miRNAs in tobacco. [file 12864_2019_6236_MOESM1_ESM.pdf]

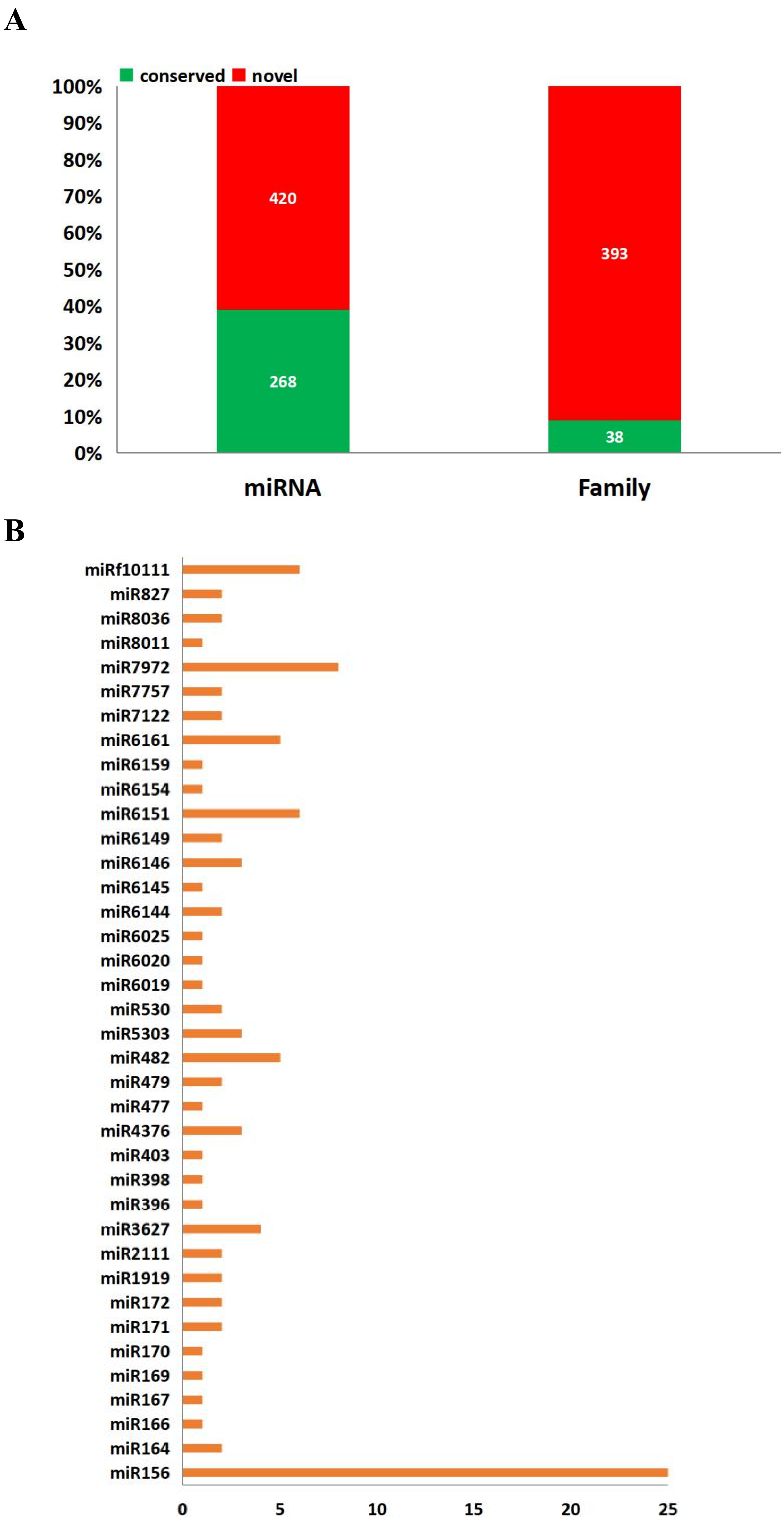

Fig. S1. Genome-wide identification of miRNAs in tobacco. (**A**) Number of conserved and novel miRNAs identified in this study. (**B**) Number of conserved miRNAs in each family.
