# Supplementary material for: A transcriptomic profile of topping responsive non-coding RNAs in tobacco roots (Nicotiana tabacum)
Source: BMC Genomics. 2019 Nov 14;20:856. doi: 10.1186/s12864-019-6236-6 (PMC6854694; doi:10.1186/s12864-019-6236-6)
Supplement: Supplementary file 2 — Additional file 2: Figure S2. Genome-wide identification of lncRNAs in tobacco. [file 12864_2019_6236_MOESM2_ESM.pdf]

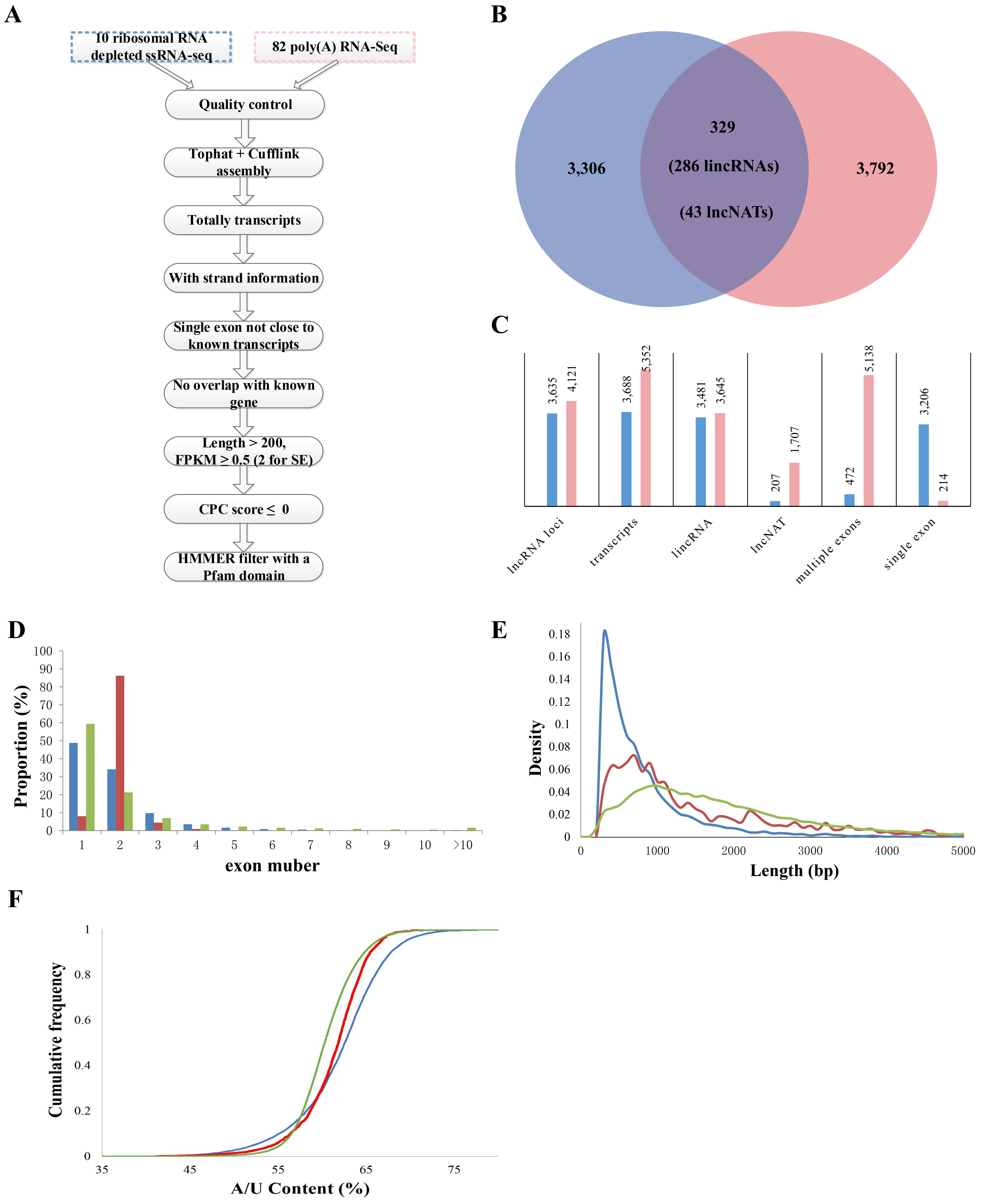

Fig. S2. Genome-wide identification of lncRNAs in tobacco. (**A**) Steps of the pipeline used in identification of lncRNAs from 10 rRNA removed strand specific RNAseq (ssRNA-seq) datasets generated in this study and 82 poly(A) RNA-seq datasets downloaded from NCBI. (**B**) Comparison of lncRNAs identified in ssRNA-seq and poly(A) RNA-seq datasets. The blue color represents result from the 10 ssRNA-seq datasets; the pink color represents result from the 82 ploy (A) RNA-seq datasets. (**C**) Number of lncRNA loci, transcripts, lincRNA, lncNAT and exons from the two types of RNA-seq datasets. (**D**) The distribution of lincRNA, lncNAT and mRNA with the exon number showing in the X-axis. (**E**) The transcript length distribution of lincRNA, lncNAT and mRNA. (**F**) A/U content of lincRNA, lncNAT and mRNA.
