# Supplementary material for: A transcriptomic profile of topping responsive non-coding RNAs in tobacco roots (Nicotiana tabacum)
Source: BMC Genomics. 2019 Nov 14;20:856. doi: 10.1186/s12864-019-6236-6 (PMC6854694; doi:10.1186/s12864-019-6236-6)
Supplement: Supplementary file 3 — Additional file 3: Figure S3. Features of circRNAs identified in tobacco and compared with other plants. [file 12864_2019_6236_MOESM3_ESM.pdf]

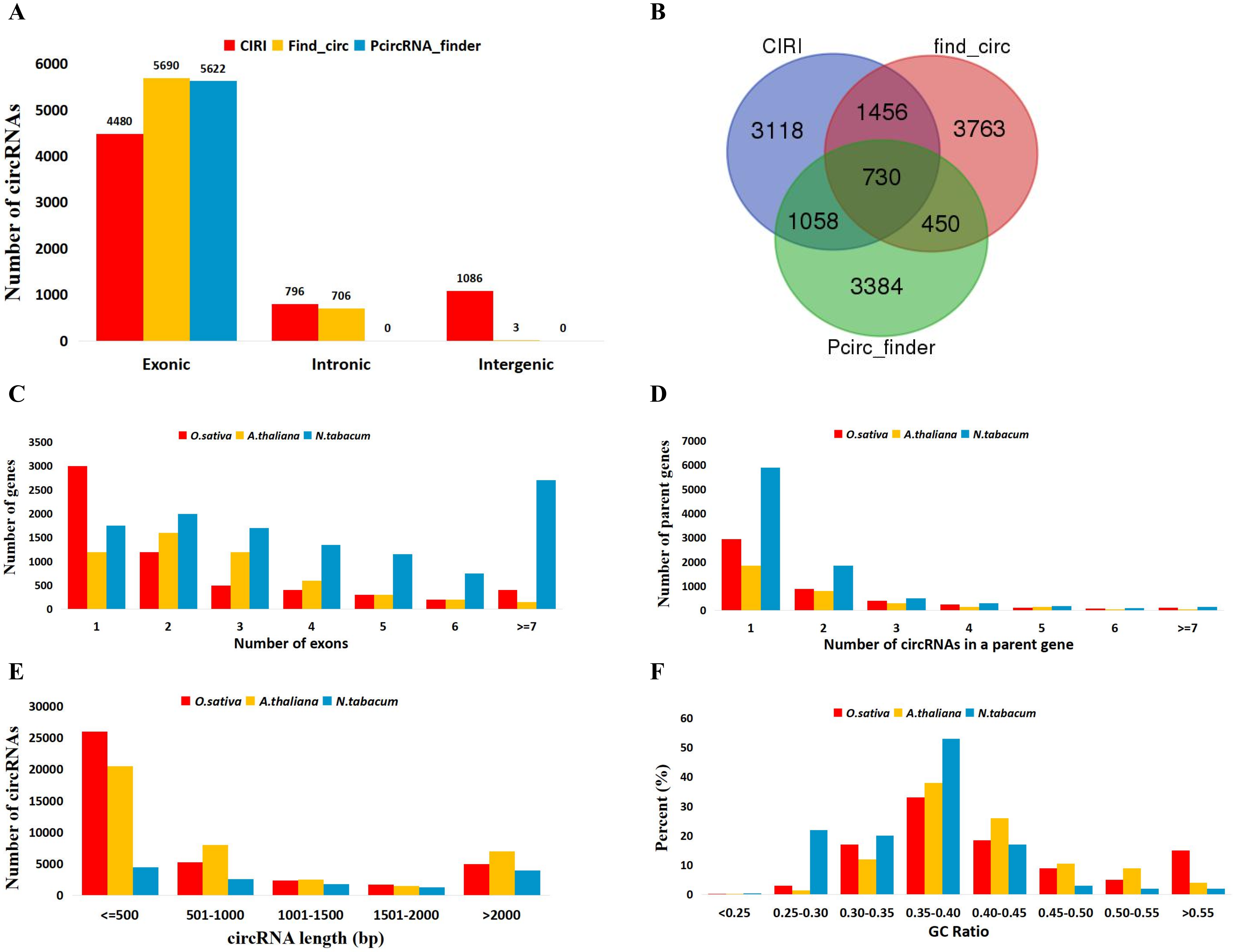

Fig. S3. Genome-wide identification of circRNAs in tobacco. (**A**) Number of different types of circRNAs identified in this study. (**B**) Number of circRNAs identified by different methods in tobacco. (**C**) Number of exonic circRNAs that contained different number of exons derived from parental genes in plants. (**D**) Number of parent genes that generated different number of circRNAs in plants. (**E**) Distribution of length of circRNAs in plants. (**F**) Distribution of GC ratio of circRNAs in plants.
