# Supplementary material for: A transcriptomic profile of topping responsive non-coding RNAs in tobacco roots (Nicotiana tabacum)
Source: BMC Genomics. 2019 Nov 14;20:856. doi: 10.1186/s12864-019-6236-6 (PMC6854694; doi:10.1186/s12864-019-6236-6)
Supplement: Supplementary file 4 — Additional file 4: Figure S4. Co-expression patterns of coding RNAs and three types of non-coding RNAs. [file 12864_2019_6236_MOESM4_ESM.pdf]

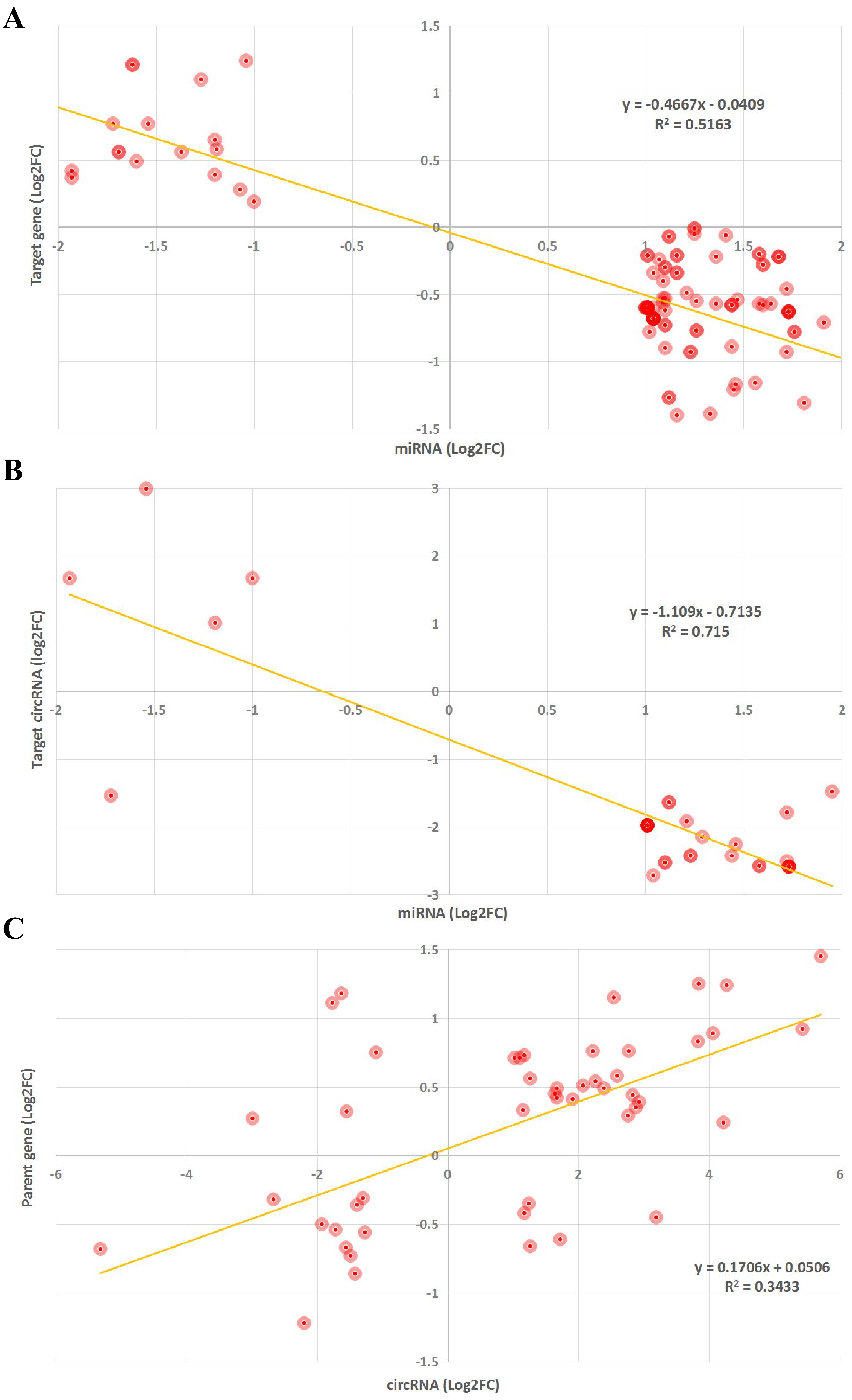

Fig. S4. Co-expression patterns of coding RNAs and three type of non-coding RNAs. (**A**) Equation of linear regression of fold-changes on log2-scale of the miRNAs (|log2FC|≥1) and their target genes. (**B**) Equation of linear regression of fold-changes on log2-scale of the miRNAs (|log2FC|≥1) and their target exonic-circRNAs (|log2FC|≥1). (**C**) Equation of linear regression of fold-changes on log2-scale of the circRNAs (|log2FC|≥1) and their host genes (FDR<0.05).
